# Non-HIV Vaccine-Induced Immune Responses as Potential Baseline Immunogenicity Predictors of ALVAC-HIV and AIDSVAX B/E-Induced Immune Responses

**DOI:** 10.3390/v16091365

**Published:** 2024-08-27

**Authors:** Ying Huang, Shomoita Alam, Erica Andersen-Nissen, Lindsay N. Carpp, One B. Dintwe, Britta S. Flach, Nicole Grunenberg, Fatima Laher, Stephen C. De Rosa, Guido Ferrari, Craig Innes, Linda-Gail Bekker, James G. Kublin, M. Juliana McElrath, Georgia D. Tomaras, Glenda E. Gray, Peter B. Gilbert

**Affiliations:** 1 Vaccine and Infectious Disease Division, Fred Hutchinson Cancer Center, Seattle, WA 98109, USA; yhuang@fredhutch.org (Y.H.); salam@fredhutch.org (S.A.); eanderse@hcrisa.org.za (E.A.-N.); lcarpp@fredhutch.org (L.N.C.); ngrunenb@fredhutch.org (N.G.); sderosa@fredhutch.org (S.C.D.R.); jkublin@fredhutch.org (J.G.K.); jmcelrat@fredhutch.org (M.J.M.); 2 Public Health Sciences Division, Fred Hutchinson Cancer Center, Seattle, WA 98109, USA; 3 Department of Biostatistics, University of Washington, Seattle, WA 98195, USA; 4 Cape Town HVTN Immunology Laboratory, Cape Town 8001, South Africa; britta_f@yahoo.com; 5 Perinatal HIV Research Unit, Faculty of Health Sciences, University of the Witwatersrand, Soweto, Johannesburg 2193, South Africa; laherf@phru.co.za; 6 Department of Laboratory Medicine and Pathology, University of Washington, Seattle, WA 98195, USA; 7 Department of Surgery, Duke University, Durham, NC 27705, USA; gflmp@duke.edu (G.F.); gdt@duke.edu (G.D.T.); 8 Duke Human Vaccine Institute, Durham, NC 27710, USA; 9 Center for Human Systems Immunology, Durham, NC 27701, USA; 10 The Aurum Institute, Klerksdorp 2570, South Africa; cinnes@auruminstitute.org; 11 The Desmond Tutu HIV Centre, University of Cape Town, Cape Town 7925, South Africa; linda-gail.bekker@hiv-research.org.za; 12 Department of Medicine, University of Washington, Seattle, WA 98195, USA; 13 Department of Integrative Immunobiology, Duke University Medical Center, Durham, NC 27710, USA; 14 Department of Molecular Genetics and Microbiology, Duke University Medical Center, Durham, NC 27710, USA; 15 South African Medical Research Council, Cape Town 7460, South Africa; glenda.gray@mrc.ac.za

**Keywords:** binding antibodies, biomarkers, HIV vaccine, immune correlates, T-cell responses

## Abstract

Identifying correlations between immune responses elicited via HIV and non-HIV vaccines could aid the search for correlates of HIV protection and increase statistical power in HIV vaccine-efficacy trial designs. An exploratory objective of the HVTN 097 phase 1b trial was to assess whether immune responses [focusing on those supported as correlates of risk (CoR) of HIV acquisition] induced via the RV144 pox-prime HIV vaccine regimen correlated with those induced via tetanus toxoid (TT) and/or hepatitis B virus (HBV) vaccines. We measured TT-specific and HBV-specific IgG-binding antibody responses and TT-specific and HBV-specific CD4+ T-cell responses at multiple time points in HVTN 097 participants, and we assessed their correlations at peak time points with HIV vaccine (ALVAC-HIV and AIDSVAX B/E)-induced responses. Four correlations were significant [false discovery rate-adjusted *p*-value (FDR) ≤ 0.2]. Three of these four were with IgG-binding antibody responses to TT measured one month after TT receipt, with the strongest and most significant correlation [rho = 0.368 (95% CI: 0.096, 0.588; *p* = 0.008; FDR = 0.137)] being with IgG-binding antibody responses to MN gp120 gDneg (B protein boost) measured two weeks after the second ALVAC-HIV and AIDSVAX B/E boost. The fourth significant correlation [(rho = 0.361; 95% CI: 0.049, 0.609; *p* = 0.021; FDR = 0.137)] was between CD4+ T-cell responses to a hepatitis B surface antigen peptide pool, measured 2 weeks after the third HBV vaccination, and IgG-binding antibody responses to gp70BCaseAV1V2 (B V1V2 immune correlate), measured two weeks after the second ALVAC-HIV and AIDSVAX B/E boost. These moderate correlations imply that either vaccine, TT or HBV, could potentially provide a moderately useful immunogenicity predictor for the ALVAC-HIV and AIDSVAX B/E HIV vaccine regimen.

## 1. Introduction

HIV vaccinology would benefit from the identification of vaccine-induced immune responses as surrogate markers for vaccine protection against HIV acquisition [1,2]. A validated biomarker, termed a correlate of protection (CoP), permits assessing surrogate vaccine protection in smaller and shorter immunogenicity studies, allowing bridging efficacy conclusions beyond resource-intensive efficacy trials [3,4]. Moreover, CoPs allow the efficient, iterative refinement of a partially efficacious vaccine. Such a CoP would be measured soon after the administration of the study vaccine so that it could be used to predict prevention efficacy. The discovery of CoPs is generally preceded by the identification of correlates of risk (CoRs) among vaccine recipients. CoRs are immune biomarkers that correlate well with the incidence of the clinical endpoint of interest. As opposed to a CoP, a CoR may or may not predict vaccine efficacy because it may merely correlate with an intrinsic factor (such as innate immunity or host genetics) that determines whether individuals are more or less naturally resistant to acquiring that clinical endpoint [1,2]. Therefore, further assessment—downstream of CoR identification, and in the context of efficacy trials—is required to validate an immune marker as a CoP.

Multiple frameworks exist for linking vaccine efficacy to an immune marker [5,6,7]. In this work, we considered the principal surrogate framework [8,9]. In this framework, the assessment of a correlation between vaccine efficacy and the level of the immune marker being examined as a candidate CoP requires the estimation of vaccine efficacy at different levels of the immune response marker. However, since placebo recipients in an HIV vaccine efficacy trial are not vaccinated, their immune response level if they were to receive vaccination is unknown [10] and needs to be predicted. The identification of a biomarker measured at the baseline—a “baseline immunogenicity predictor”—that can well predict a vaccine-induced immune CoR can be invaluable in assessing whether the CoR is also a CoP.

After the RV144 trial (NCT00223080) demonstrated modest (31.2% at 42 months) vaccine efficacy against HIV acquisition in its modified-intention-to-treat analysis of the ALVAC-HIV and AIDSVAX B/E vaccine regimen [11], immune correlates were identified [12]: IgG-binding antibodies to HIV Env V1V2 as an inverse CoR and plasma IgA-binding antibodies to Env as a direct CoR [13]. Secondary analyses in the same work identified four further correlates: Env-specific IgA antibodies with IgG avidity, antibody-dependent cellular cytotoxicity (ADCC), neutralizing antibodies, and CD4+ T cells, as an inverse CoR when in concert with low levels of plasma IgA-binding antibodies to Env [13]. Follow-up studies supported vaccine-elicited ADCC as potentially contributing to vaccine protection [14]. Other immune correlates analyses of the RV144 trial supported IgG-binding antibodies to Env V1V2 [15] and identified additional CoRs, including Env-specific CD4+ T cell polyfunctionality [16], IgG3-binding antibodies to Env V1V2 [17], and IgG-binding antibodies to gp120 V2 and V3 [18]. Identifying baseline factors that correlate with these CoRs would contribute to knowledge about whether these CoRs are also CoPs.

Little work has evaluated whether external immune parameters correlate with HIV vaccine-induced immune responses. However, some associations have been identified: age, sex at birth, and BMI were all found to be associated with the HIV-specific CD4+ T-cell response rate after receipt of a DNA plasmid HIV vaccine [19]; sex assigned at birth, heavy drinking of alcohol, and BMI were found to be associated with clade C antigen-specific interferon-γ ELISPOT responses in the Phambili study of a MRK Ad5 HIV gag/pol/nef subtype B vaccine; baseline adenovirus 5 (Ad5) titers were found to be associated with HIV-specific interferon–ELISPOT responses in the Step trial of the MRKAd5 HIV gag/pol/nef vaccine [20]; the baseline Ad5 titer was found to be negatively associated with HIV-specific CD8+ responses after the receipt of a DNA prime/rAd5 boost regimen [21].

The primary objectives of the HVTN 097 trial (NCT02109354) have been reported, evaluating the safety and immunogenicity of the RV144 HIV vaccine regimen (two administrations of ALVAC-HIV followed by two co-administrations of ALVAC-HIV and AIDSVAX B/E) in South Africa [22]. The exploratory objectives included assessing the association of HIV vaccine-induced immune responses with immune responses induced via two different licensed vaccines [tetanus toxoid (TT) and hepatitis B virus (HBV)]. The rationale of this objective originated in part from the findings of Czeschinski et al., who reported that anti-hepatitis A virus (HAV) antibody titer correlated with anti-hepatitis B surface antigen (HBsAg) antibody titer (measured one month after the final dose)—whether the HBV and HAV vaccines were separately or co-administered [23].

In this work, we report humoral and cellular immune responses induced via the TT and HBV vaccines in the HVTN 097 trial, study the association of these TT-specific and HBV-specific responses, and assess whether immune responses to either vaccine correlated with those induced via the ALVAC-HIV and AIDSVAX B/E vaccine regimen.

## 2. Materials and Methods

### 2.1. HVTN 097 Trial and Vaccine Regimen

The HVTN 097 trial was a phase-1b randomized, double-blind, placebo-controlled clinical trial that found through its primary objective that the RV144 vaccine regimen was safe and immunogenic in participants in South Africa. The trial was registered with the U.S. National Institutes of Health (NIH) Clinical Trials Registry (ClinicalTrials.gov NCT02109354) and the South African National Clinical Trials Registry (SANCTR number: DOH-27-0313-4201). Eligible participants (*n* = 100) without HIV, aged 18 to 40 years, with no history of tetanus who were negative for HBsAg, were enrolled at three sites in South Africa [22]. South Africa introduced into the expanded program of immunization the TT vaccine in 1938 and hepatitis B vaccination in infants in April 1995. Given that HBV coverage among 1-year-olds in South Africa in 2010 was 71% [24], it was estimated that, by 2013, the year HVTN 097 began recruitment and enrollment, most 18-year-olds would not have received hepatitis B immunization.

**Figure 1:**
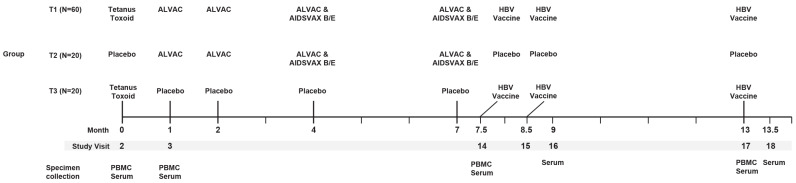
Schema of the HVTN 097 trial, along with sampling time points.

Written, informed consent was obtained from all participants. There were two active comparator arms (T1 and T2) and one placebo comparator arm (T3) (Figure 1). The participants were randomized (3:1:1) to T1, T2, or T3. The participants in T1 (*n* = 60) received the TT vaccine at Month 0, ALVAC-HIV at Months 1 and 2, AIDSVAX B/E at Months 4 and 7, and the hepatitis B vaccine (HBV) at Months 7.5, 8.5, and 13. Participants in T2 (*n* = 20) received a placebo injection at Month 0, ALVAC-HIV at Months 1 and 2, AIDSVAX B/E at Months 4 and 7, and placebo injections at Months 7.5, 8.5, and 13. Participants in T3 (*n* = 20) received the TT vaccine at Month 0, placebo injections at Months 1, 2, 4, and 7, and HBV at Months 7.5, 8.5, and 13. In the text, we refer to these regimens as T1_TT-HIV-HBV_, T2_HIV_, and T3_TT-HBV_.

### 2.2. Selection of HIV Vaccine-Induced Immune Responses to Be Assessed for Correlations with Non-HIV Vaccine-Induced Immune Responses

HIV vaccine-induced immune responses that were assessed for correlations with non-HIV vaccine-induced immune responses were selected based on the existence of previous evidence as a CoR of HIV acquisition [13,16,17,22,25,26], and they are shown in Table S1.

### 2.3. Intracellular Cytokine Staining (ICS)

Flow cytometry was used to examine TT-specific and HBsAg-specific CD4+ and CD8+ T-cell responses using a validated ICS assay [27] involving the stimulation of PBMC with tetanus toxoid (Calbiochem, San Diego, CA, USA) or a synthetic peptide pool covering HBsAg (lot number 15F046A; Bio-Synthesis Incorporated, Lewisville, TX, USA). HIV vaccine-specific CD4+ T-cell responses to 92TH023-Env and LAI-Gag peptide pools were assessed on a LSR Fortessa Cell Analyser (BD Biosciences, Franklin Lakes, NJ, USA) as previously described [13,16,28]. The gating strategy is shown in Figure S1.

The response magnitude was defined as the percentage of total CD4+ or CD8+ T cells expressing intracellular IFN-gamma and/or IL-2 following ex vivo stimulation with the antigens listed above. Positivity calls for individual cytokines or cytokine combinations were determined via a one-sided Fisher’s exact test applied to each antigen-specific response versus the negative control response with a discrete Bonferroni adjustment for the multiple comparisons due to testing for responses to multiple antigens. Antigen responses with adjusted *p*-values of less than 0.00001 were considered positive. If at least one peptide pool for a specific HIV protein was positive, then the overall response to the protein was considered positive.

The polyfunctionality score (for HIV-specific CD4+ T-cell responses) was computed based on the expression of IFN-γ, IL-2, TNF, IL-4, and CD154 in the ICS assay using the COMPASS method [16].

### 2.4. IgG- and IgA-Binding Antibody Responses

TT and HBV-specific IgG and HIV-specific IgG- and IgA-binding antibody responses in serum were assessed via a binding antibody multiplex assay (BAMA), as described [13,15,17,28,29]. Antigens utilized in the assays for TT and Hepatitis B are TT-RP (reagent proteins) and the HBsAg full-length protein (Abcam, Cambridge, UK). The specific antigens against which HIV-specific IgG- and IgA-binding antibody responses were assessed are provided in Table S1. The readout is background-subtracted mean fluorescence intensity (MFI), where the background refers to a plate-level control (i.e., a blank well run on each plate). Samples from post-enrollment visits were declared to have positive responses if they met three conditions: (1) the MFI minus blank values were ≥ the antigen-specific cutoff (based on the average + 3 standard deviations of 60 seronegative plasma samples), (2) the MFI minus blank values were greater than 3 times the baseline (day 0) MFI minus blank values, and (3) the MFI values (not background-subtracted) were greater than 3 times the baseline MFI values (not background-subtracted).

### 2.5. Antibody-Dependent Cellular Cytotoxicity (ADCC) Responses

ADCC assays were performed as described [22,30]. ADCC-mediated antibody responses were measured using an ADCC GranToxiLux (GTL) assay kit (OncoImmunin, Inc., Gaithersburg, MD, USA) and quantified as net-percent granzyme B activity, which is the percentage of target cells positive for GTL detected via flow cytometry, measured at six serum dilutions: 50, 250, 1250, 6250, 31,250, and 156,256 for each antigen. The gating strategy has been previously published, and it is shown in Figure 1B of Pollara et al. [30]. Nonparametric-area-under-the-net-percentage granzyme B activity vs. the Log10 (dilution) curve (“AUC”) was calculated using the trapezoidal rule.

### 2.6. Antibody-Dependent Cellular Phagocytosis (ADCP) Responses

ADCP assays were performed as described [22]. The quantification of ADCP was performed for covalently binding clade C 1086 gp140 envelope glycoprotein to fluorescent beads and forming immune complexes via incubation in the presence of a 1:50 dilution of serum. Immune complexes were then incubated in the presence of monocyte THP-1 cells (ATCC, Manassas, VA, USA), and the fluorescence of the cells was detected using flow cytometry (BD LSRFortessa, BD Biosciences, Franklin Lakes, NJ, USA). ADCP scores were calculated by multiplying the MFI and frequency of phagocytosis-positive cells and then dividing by the MFI and frequency of phagocytosis-positive cells in an antibody-negative (PBS) control well. Representative FACS plots are shown by Tay et al. [31].

### 2.7. Statistical Methods

Boxplots are used to graphically display the distribution of immune responses to individual antigens at each given time point. The response rate for an immune response was computed as the percentage of participants who had a positive response (as defined in Section 2.3 and Section 2.4 for ICS and BAMA, respectively). To compare response rates and magnitudes among positive responders between two independent groups, Fisher’s exact test and the Wilcoxon rank sum test were used, respectively. All *p*-values are two-sided.

Scatterplots are used to display pairwise relationships between (i) TT-specific immune responses and HBV-specific immune responses, and (ii) non-HIV vaccine-specific immune responses and selected HIV vaccine-specific CoRs from the five HIV immune response categories (IgG-binding antibody, IgA-binding antibody, ADCC, ADCP, and CD4+ T-cell). All correlation analyses for (i) and (ii) were performed using data from the T1_TT-HIV-HBV_ arm only, in case HIV vaccination affected any potential correlation between TT-induced and HBV vaccine-induced immune responses. The correlations shown in all plots are Spearman’s rank correlations (rho). Multiplicity adjustment was applied across pairs of immune responses with the false discovery rate (FDR) computed using the Benjamini–Hochberg procedure [32] to assess the correlation between TT and HBV immune responses, as well as the correlation between non-HIV vaccine-specific immune response and each HIV CoR category separately. Significant correlations were defined according to FDR < 0.2.

All statistical analyses were done in R (version 4.1.1) [33]. The stats package [34] was used for the correlation tests and plotting.

## 3. Results

### 3.1. Participant Baseline Characteristics

Of the enrolled participants, 51% were assigned a male sex at birth, and 100% were Black African. The median age was 21.5 years [inter-quartile range (IQR): 20–25 years].

### 3.2. TT-Specific Immune Responses

#### 3.2.1. CD4+ T-Cell Responses

**Figure 2:**
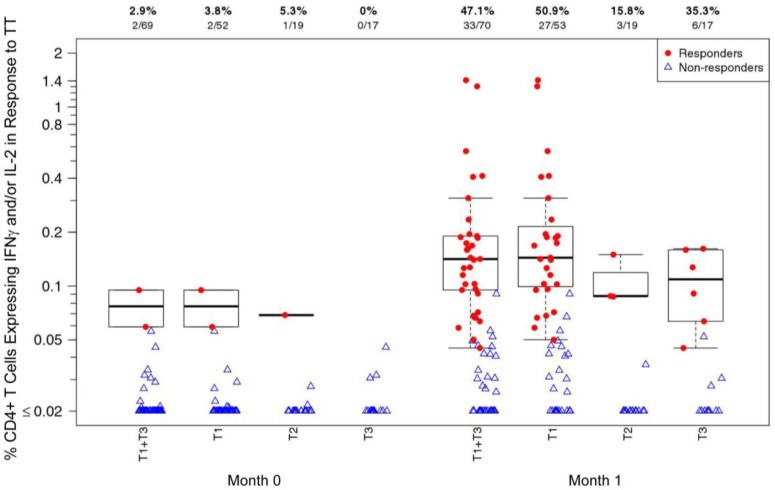
CD4+ T-cell responses (% CD4+ T cells expressing IFNγ and/or IL-2) to tetanus toxoid (TT) at Month 0 and at Month 1 [1 month after TT vaccination; T1_TT-HIV-HBV_ and T3_TT-HBV_] in HVTN 097 participants, stratified by treatment assignment arm. At Month 0, T1_TT-HIV-HBV_ and T3_TT-HBV_ received TT; T2_HIV_ received a placebo. Response rates and numbers of participants are shown at the top of the plot. Responders: red dot; non-responders: open blue triangle.

CD4+ T-cell response rates and magnitudes (% CD4+ T cells expressing IFNγ and/or IL-2) to TT at the baseline (Month 0) and at one month after TT vaccination [Month 1; T1_TT-HIV-HBV_, T3_TT-HBV_] are shown in Figure 2. Both TT-vaccinated groups [T1_TT-HIV-HBV_ and T3_TT-HBV_] showed an increase in CD4+ T-cell responses to TT at Month 1 compared to Month 0. The T1_TT-HIV-HBV_+T3_TT-HBV_ combined response rate was 2.9% at Month 0, which increased to 47.1% at Month 1. Group T2_HIV_, who received a placebo instead of TT at Month 0, also showed an increase in their CD4+ T-cell response rate to TT from 3.8% (Month 0) to 15.8% (Month 1). This increase was due to two low-level positive responders at Month 1. There were no significant differences in response rates (Fisher’s exact test) or the magnitudes of positive responses (Wilcoxon rank sum test) between groups T1_TT-HIV-HBV_ and T3_TT-HBV_; however, the response rate was greater in T1_TT-HIV-HBV_+T3_TT-HBV_ than in T2_HIV_ at Month 1 (*p* = 0.02). Two outliers in the T1_TT-HIV-HBV_ group were observed with high CD4+ T-cell response magnitudes. Of these two outliers, only one had a positive response at Month 0. The responder with the highest magnitude (1.41%) at Month 1 also had the highest magnitude at Month 0 (0.09%) and had a positive response at both months. The other outlier (1.30%) had a negative response at Month 0. No CD8+ T-cell responses were detected at either time point in any group.

#### 3.2.2. IgG-Binding Antibody Responses

**Figure 3:**
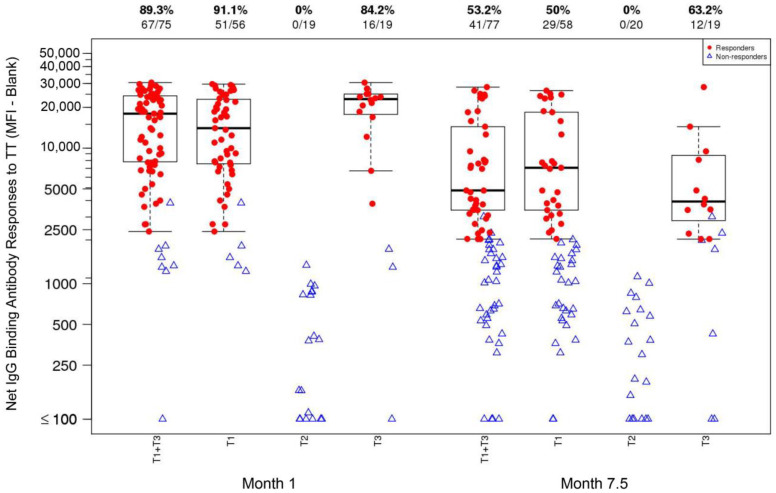
Net IgG-binding antibody responses (MFI-blank) to tetanus toxoid (TT) (1:2000 dilution) at Month 1 [1 month after TT vaccination; T1_TT-HIV-HBV_ and T3_TT-HBV_] and at Month 7.5 [2 weeks after the last ALVAC and AIDSVAX B/E vaccination; T1_TT-HIV-HBV_ and T2_HIV_] in the HVTN 097 participants, stratified by treatment assignment arm. At Month 0, T1_TT-HIV-HBV_ and T3_TT-HBV_ received TT; T2_HIV_ received a placebo. At Month 7, T1_TT-HIV-HBV_ and T2_HIV_ received the last dose of ALVAC and AIDSVAX B/E; T3_TT-HBV_ received a placebo. Response rates and numbers of participants are shown at the top of the plot. Responders: red dot; non-responders: open blue triangle.

IgG-binding antibody response rates and magnitudes in response to TT (1:2000 dilution) at Month 1 [1 month after TT vaccination; T1_TT-HIV-HBV_ and T3_TT-HBV_] and at Month 7.5 [2 weeks after the last ALVAC and AIDSVAX B/E vaccination; T1_TT-HIV-HBV_ and T2_HIV_] are shown in Figure 3. The response rates at Month 1 and Month 7.5 were 91.1% and 50%, respectively, in T1_TT-HIV-HBV_ and 84.2% and 63.2%, respectively, in T3_TT-HBV_. The responses in T2_HIV_ were 0% across both time points. There were no significant differences in response rates between T1_TT-HIV-HBV_ and T3_TT-HBV_, and the pooled T1_TT-HIV-HBV_+T3_TT-HBV_ response rate was significantly higher than that of T2_HIV_ at each time point (*p* < 0.0001 for both Month 1 and Month 7.5).

### 3.3. HBV-Specific Immune Responses

#### 3.3.1. CD4+ T-Cell Responses

**Figure 4:**
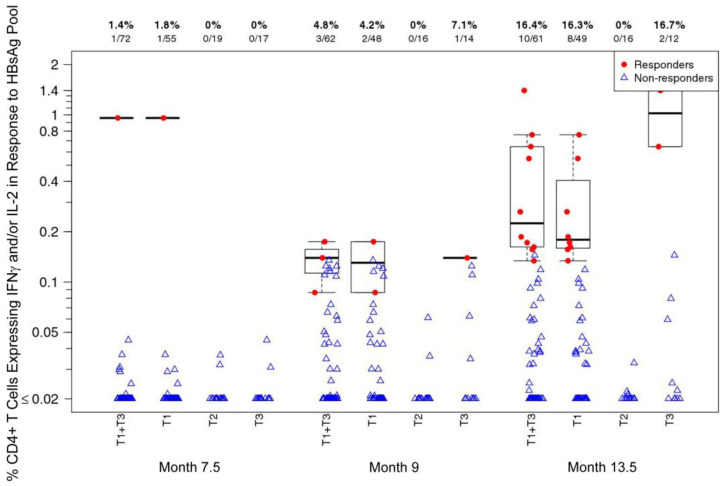
CD4+ T-cell responses (% CD4+ T cells expressing IFNγ and/or IL-2) to an HBsAg peptide pool at Month 7.5 and Month 9 [2 weeks after the second hepatitis B virus (HBV) vaccination; T1_TT-HIV-HBV_ and T3_TT-HBV_], and Month 13.5 [2 weeks after the third HBV vaccination; T1_TT-HIV-HBV_ and T3_TT-HBV_] in the HVTN 097 participants, stratified by treatment assignment arm. At Months 7.5, 8.5, and 13, T1_TT-HIV-HBV_ and T3_TT-HBV_ received HBV; T2_HIV_ received a placebo. Response rates and numbers of participants are shown at the top of the plot. Responders: red dot; non-responders: open blue triangle.

At Month 7.5, i.e., the baseline for assessing HBV-specific immune responses, the combined T1_TT-HIV-HBV_+T3_TT-HBV_ group CD4+ T-cell response rate to an HBsAg peptide pool was 1.4%. This response rate increased to 4.8% two weeks after the second HBV vaccination (Month 9) and further increased to 16.4% two weeks after the third HBV vaccination (Month 13.5) (Figure 4). The T1_TT-HIV-HBV_+T3_TT-HBV_ combined group response rate at Month 13.5 was significantly higher than at Month 7.5 (*p* = 0.0067). There were no significant differences between the CD4+ T-cell response rates or magnitudes between the T1_TT-HIV-HBV_ and T3_TT-HBV_ groups at Months 7.5, 16, or 18. There were no responders in the T2_HIV_ group (received placebo at Months 7.5, 8.5, and 13) at Months 7.5, 16, or 18. The T1_TT-HIV-HBV_+T3_TT-HBV_ combined group response rates were significantly higher at v18 compared to v14 (*p* = 0.0067). There were no significant differences between the CD4+ T-cell response rates or magnitudes between the T1_TT-HIV-HBV_ and T3_TT-HBV_ groups at any time point.

#### 3.3.2. IgG-Binding Antibody Responses

**Figure 5:**
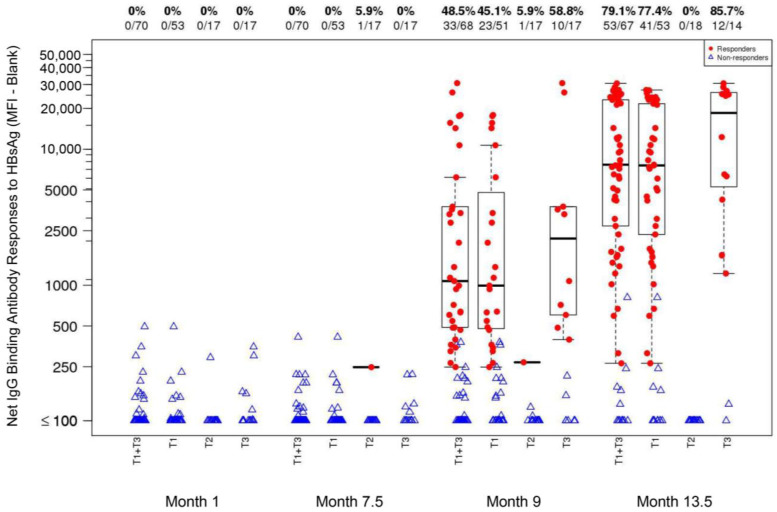
Net IgG-binding antibody responses [mean fourescence intensity (MFI)-blank] to HBsAg (1:50 dilution) at Month 1 [1 month after TT vaccination; T1_TT-HIV-HBV_ and T3_TT-HBV_], Month 7.5 [two weeks after the last ALVAC and AIDSVAX B/E vaccination; T1_TT-HIV-HBV_ and T2_HIV_], Month 9 [2 weeks after the second HBV vaccination; T1_TT-HIV-HBV_ and T3_TT-HBV_], and Month 13.5 [2 weeks after the third HBV vaccination; T1_TT-HIV-HBV_ and T3_TT-HBV_] in the HVTN 097 participants, stratified by treatment assignment arm. At Month 0, T1_TT-HIV-HBV_ and T3_TT-HBV_ received TT; T2_HIV_ received a placebo. At Months 7.5, 8.5, and 13, T1_TT-HIV-HBV_ and T3_TT-HBV_ received HBV; T2_HIV_ received a placebo. Response rates and numbers of participants are shown at the top of the plot. Responders: red dot; non-responders: open blue triangle.

IgG-binding antibody response rates to HBsAg at Months 1, 7.5, 9, and 13.5 were 0%, 0%, 45.1%, and 77.4% in T1_TT-HIV-HBV_ and 0%, 0%, 58.8%, and 85.7% in T3_TT-HBV_, respectively. There were no significant differences between the T1_TT-HIV-HBV_ and T3_TT-HBV_ response rates, and the pooled T1_TT-HIV-HBV_+T3_TT-HBV_ response rate was significantly higher than the T2_HIV_ response rate at every time point (Figure 5). In T2_HIV_ (received placebo at Months 7.5, 8.5, and 13), the response rates were lower than 6% across all four time points (Months 1, 7.5, 9, and 13.5).

### 3.4. Correlations between TT-Induced and HBV-Induced Immune Responses at Peak Time Points

**Figure 6:**
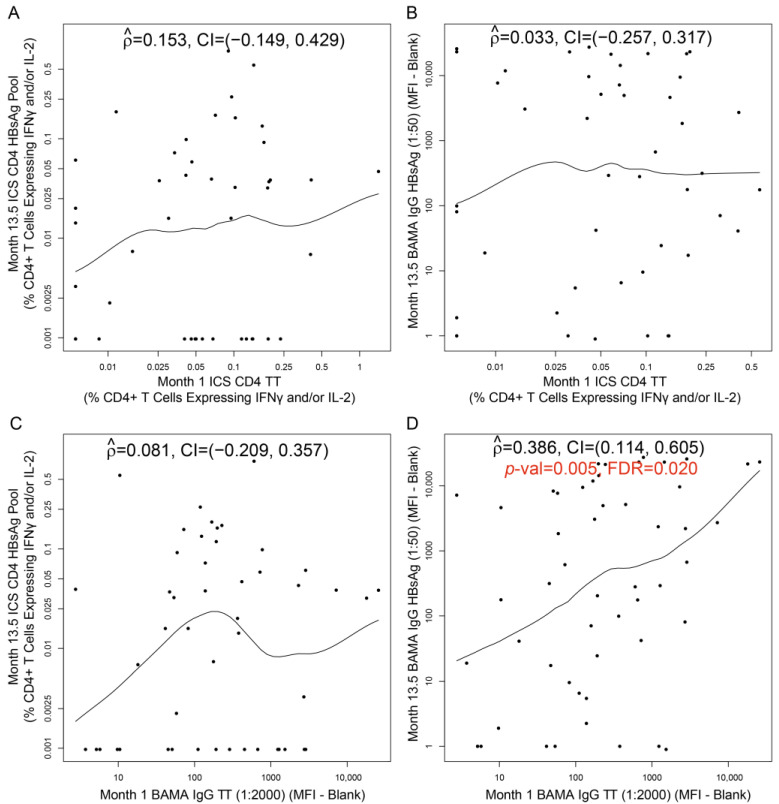
Correlations in HVTN 097 participants [T1_TT-HIV-HBV_ arm only] for (**A**) Month 1 TT-specific CD4+ T-cell responses versus Month 13.5 HBsAg-specific CD4+ T-cell responses; (**B**) Month 1 TT-specific CD4+ T-cell responses versus Month 13.5 HBV-specific BAMA IgG responses; (**C**) Month 1 TT-specific BAMA IgG responses versus Month 13.5 HBsAg-specific CD4+ T-cell responses; (**D**) Month 1 TT-specific BAMA IgG responses versus Month 13.5 HBV-specific BAMA IgG responses. ICS CD4: CD4+ T-cell responses (% CD4+ T cells expressing IFNγ and/or IL-2) to the designated peptide pool; BAMA IgG: Net IgG-binding antibody responses (MFI-blank) to the designated antigen. Each dot represents one participant. At Month 0, T1_TT-HIV-HBV_ participants received TT. At Months 7.5, 8.5, and 13), T1_TT-HIV-HBV_ participants received HBV. For the correlation with FDR ≤ 0.2, the *p*-value and false discovery rate (FDR) value are shown in red font (panel D). For all other panels, the correlation was not significant (FDR > 0.2).

We next examined whether TT-specific binding antibody and/or TT-specific CD4+ T-cell responses at Month 1 [1 month after TT vaccination; T1_TT-HIV-HBV_] correlated with HBV-specific binding antibody and/or HBV-specific CD4+ T-cell responses at Month 13.5 [2 weeks after the third HBV vaccination; T1_TT-HIV-HBV_] (Figure 6). In general, TT-induced and HBV-induced immune responses at peak time points were not correlated, with the exception of a weak-to-moderate correlation [rho = 0.386, confidence interval (CI): 0.114, 0.605; *p* = 0.005, FDR = 0.020, thus passing the prespecified threshold for significance (FDR ≤ 0.2)] between Month 1 IgG-binding antibody responses to TT and Month 13.5 IgG-binding antibody responses to HBsAg in T1_TT-HIV-HBV_ participants. The fact that the TT-induced immune responses were not highly correlated with the HBV-induced immune responses lends support to investigating each of the two vaccines for potential correlations with HIV vaccine-induced responses.

### 3.5. Correlations between Non-HIV Vaccine (TT, HBV)-Induced Immune Responses and HIV Vaccine-Induced Immune Responses at Peak Time Points

**Figure 7:**
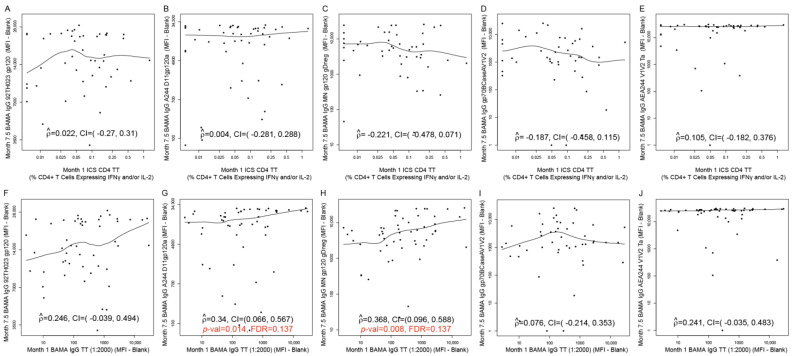
Correlation plots for (**A**–**E**) Month 1 TT-specific CD4+ T-cell responses versus Month 7.5 IgG-binding antibody responses against the HIV antigen designated on each *y*-axis; (**F**–**J**) Month 1 TT-specific IgG-binding antibody responses versus Month 7.5 IgG-binding antibody responses against the HIV antigen designated on each *y*-axis. Data are shown for HVTN 097 participants [T1_TT-HIV-HBV_ arm only]. ICS CD4 TT: CD4+ T-cell responses (% CD4+ T cells expressing IFNγ and/or IL-2) to TT; BAMA IgG TT: Net IgG-binding antibody responses (MFI-blank) to TT. Each dot represents one participant. At Month 0, T1_TT-HIV-HBV_ participants received TT. At Month 7, T1_TT-HIV-HBV_ received the last dose of ALVAC and AIDSVAX B/E. For correlations with FDR ≤ 0.2, the *p*-values and FDR values are shown in red font (panels G, H). For all other panels, the correlation was not significant (FDR > 0.2).

**Figure 8:**
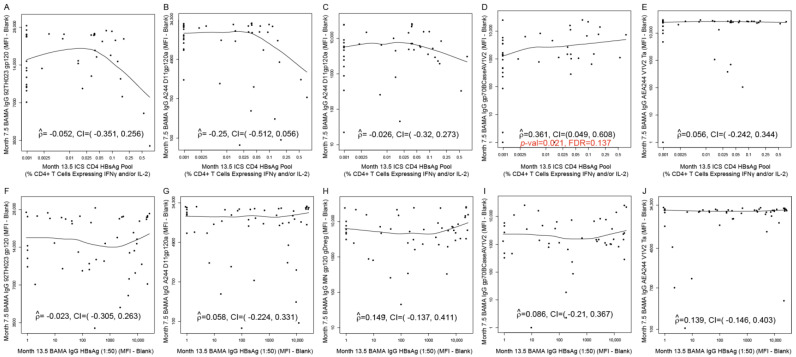
Correlation plots for (**A**–**E**) Month 13.5 HBsAg-specific CD4+ T-cell responses versus Month 7.5 IgG-binding antibody responses against the HIV antigen designated on each *y*-axis; (**F**–**J**) Month 13.5 HBsAg-specific IgG-binding antibody responses versus Month 7.5 IgG-binding antibody responses against the HIV antigen designated on each *y*-axis. Data are shown for HVTN 097 participants [T1_TT-HIV-HBV_ arm only]. ICS CD4 HBsAg pool: CD4+ T-cell responses (% CD4+ T cells expressing IFNγ and/or IL-2) to HBsAg pool; BAMA IgG HBsAg: Net IgG-binding antibody responses (MFI-blank) to HBsAg. Each dot represents one participant. At Month 13, T1_TT-HIV-HBV_ participants received HBV. At Month 7, T1_TT-HIV-HBV_ received the last dose of ALVAC and AIDSVAX B/E. For the correlation with FDR ≤ 0.2, the *p*-value and FDR value are shown in red font (panel D). For all other panels, the correlation was not significant (FDR > 0.2).

**Figure 9:**
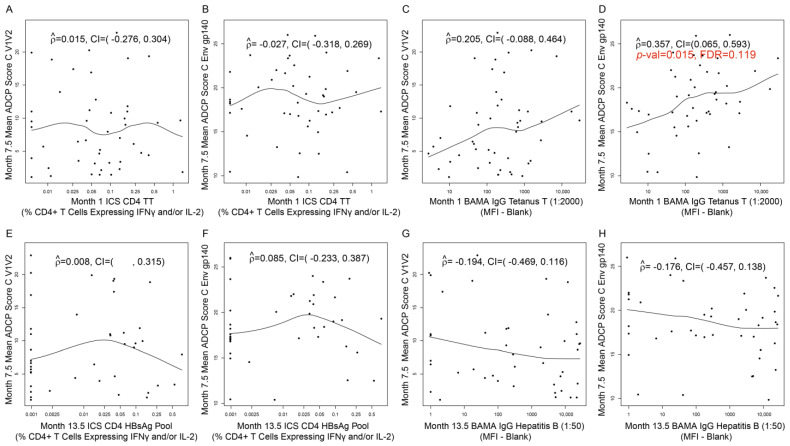
Correlation plots for (**A**,**B**) Month 1 TT-specific CD4+ T-cell responses versus Month 7.5 mean ADCP scores against the HIV antigen designated on each *y*-axis; (**C**,**D**) Month 1 TT-specific IgG-binding antibody responses versus Month 7.5 mean ADCP scores against the HIV antigen designated on each *y*-axis; (**E**,**F**) Month 13.5 HBsAg-specific CD4+ T-cell responses versus Month 7.5 mean ADCP scores against the HIV antigen designated on each *y*-axis; (**G**,**H**) Month 13.5 HBsAg-specific IgG-binding antibody responses versus Month 7.5 mean ADCP scores against the HIV antigen designated on each *y*-axis. Data are shown for HVTN 097 participants [T1_TT-HIV-HBV_ arm only]. ICS CD4 HBsAg pool: CD4+ T-cell responses (% CD4+ T cells expressing IFNγ and/or IL-2) to HBsAg pool; BAMA IgG HBsAg: Net IgG-binding antibody responses (MFI-blank) to HBsAg. Each dot represents one participant. At Month 13, T1_TT-HIV-HBV_ participants received HBV. At Month 7, T1_TT-HIV-HBV_ received the last dose of ALVAC and AIDSVAX B/E. For the correlation with FDR ≤ 0.2, the *p*-value and FDR value are shown in red font (panel D). For all other panels, the correlation was not significant (FDR > 0.2).

Correlations between non-HIV vaccine (TT and HBV)-induced immune responses (at Month 1 and Month 13.5, respectively) and HIV vaccine-induced immune responses at Month 7.5 (T1_TT-HIV-HBV_ arm) are presented for each of the five categories of HIV-vaccine CoR: IgG-binding antibodies (Figure 7 and Figure 8), IgA-binding antibodies (Figure S2), ADCC (Figure S3), ADCP (Figure 9), and CD4+ T cells (Figures S4 and S5). Of all the assessed correlations between non-HIV vaccine (TT and HBV)-induced immune responses and HIV vaccine-induced IgG-binding antibody responses, four passed the prespecified threshold for significance with FDR control within the corresponding immune response category (FDR ≤ 0.2):

1. Month 1 IgG-binding antibody responses to TT with Month 7.5 IgG-binding antibody responses to A244 D11gp120a (AE protein boost) (rho = 0.340; 95% CI: 0.066, 0.567; *p* = 0.014; FDR = 0.137) (Figure 7G);
2. Month 1 IgG-binding antibody responses to TT with Month 7.5 IgG-binding antibody responses to MN gp120 gDneg (B protein boost) (rho = 0.368; 95% CI: 0.096, 0.588; *p* = 0.008; FDR = 0.137) (Figure 7H);
3. Month 13.5 CD4+ T-cell responses to HBsAg peptide pool with Month 7.5 IgG-binding antibody responses to gp70BCaseAV1V2 (B V1V2 immune correlate) (rho = 0.361; 95% CI: 0.049, 0.609; *p* = 0.021; FDR = 0.137) (Figure 8D);
4. Month 1 IgG-binding antibody responses to TT with Month 7.5 mean ADCP score against C Env gp140 (rho = 0.357; 95% CI: 0.065, 0.593; *p* = 0.015; FDR = 0.119) (Figure 9D).

Figure 7 shows the rest of the assessed correlations between Month 1 TT-induced immune responses and Month 7.5 HIV vaccine-induced IgG-binding antibody responses. Figure 8 shows the rest of the assessed correlations between Month 13.5 HBV-induced immune responses and Month 7.5 HIV vaccine-induced IgG-binding antibody responses. Figure 9 shows the rest of the assessed correlations between Month 1 TT-induced immune responses or Month 13.5 HBV-induced immune responses and Month 7.5 HIV vaccine-induced ACDP responses. Except for the four correlations above, none of the correlations shown in these figures were significant.

Among the other categories of assessed HIV vaccine-induced immune responses (IgA-binding antibody responses, ADCC activity, and CD4+ T-cell responses), none of the correlations with tetanus-induced immune responses or with HBV-induced immune responses were significant (Figures S2–S5).

## 4. Discussion

In the HVTN 097 trial, three vaccines were evaluated: two non-HIV vaccines (TT and HBV) and the same HIV vaccine regimen shown to be partially efficacious in the Thai RV144 trial. Our analysis found that certain HIV vaccine-induced immune responses correlated with non-HIV vaccine-induced immune responses. In our analysis, we adopted the Benjamini–Hochberg procedure to control the false discovery rate given the multiple pairs of immune responses studied for their correlation. This approach was chosen over the Bonferroni adjustment procedure, a conservative technique for controlling the family-wise error rate, which is deemed overly conservative for our objective. The correlations support previous work by other groups [23] suggesting that immune responses to one vaccine could predict responses to another.

A major motivation of the present analysis is that baseline immunogenicity predictors could be used to increase the statistical power of assessing immune response biomarkers as principal surrogate CoPs. The incorporation of baseline immunogenicity predictors is one of the two approaches proposed by Follmann [10] to predict the “counterfactual” values of the vaccine-induced immunological biomarkers for placebo recipients in vaccine efficacy trials. The other approach is to augment the study design with a close-out placebo vaccination (CPV) component wherein trial participants in the placebo arm who had not acquired HIV by the conclusion of the trial are administered the HIV vaccine at that time, and their immune responses measured to substitute the unmeasured counterfactual values. Follmann compared the use of a baseline immunogenicity predictor with the use of CPV to achieve increased statistical power in a subsequent CoP assessment [10]. Table 4 in [10] compares the power of baseline immunogenicity predictor, CPV, and baseline immunogenicity predictor + CPV correlations of 0.25 and 0.5, showing that a baseline immunogenicity predictor with a correlation of 0.25 has a power similar to CPV alone, whereas the baseline immunogenicity predictor + CPV improves the power over the baseline immunogenicity predictor alone or CPV alone. When rho = 0.5, the power achieved with a baseline immunogenicity predictor is not further improved by CPV. Therefore, the significant correlations identified in the present study (rho ranging from 0.340 to 0.368) would likely have reasonable power, depending on setting, and they may also be further improved via CPV.

Three of the four significant correlations with HIV vaccine-induced immune responses were with TT-induced binding antibody responses, whereas the other was with HBV-induced CD4+ T-cell responses. The magnitudes of these correlations were all relatively similar (as stated above, rho ranging from 0.340 to 0.368), and we therefore conclude that either vaccine, TT or HBV, could potentially be used as a moderate baseline immunogenicity predictor of the ALVAC-HIV and AIDSVAX B/E vaccine regimen used in RV144. The TT vaccine was administered prior to the ALVAC-HIV and AIDSVAX B/E vaccine regimen, whereas the HBV vaccine was administered after the ALVAC-HIV and AIDSVAX B/E vaccine regimen. Thus, if the HBV vaccine-induced immune response (Month 13.5 CD4+ T-cell responses to HBsAg peptide pool) shown to correlate with the HIV vaccine-induced immune response (Month 7.5 IgG-binding antibody responses to gp70BCaseAV1V2) were to be used as a baseline immunogenicity predictor in an HIV vaccine trial (with additional caveats discussed below, including that this correlation might not be seen with other HIV vaccine regimens), it would additionally need to be verified that the two responses still correlate if the HBV vaccine is administered before the HIV vaccine regimen.

Our study had a number of limitations. First, it is unknown whether or how the moderate correlations between immune responses elicited via non-HIV and HIV vaccines identified in this work would also be observed in other populations, given that vaccine-elicited responses also vary by participant-level factors (e.g., age, genes, and previous infections) [35], as well as environmental factors [36]. Second, a prime-boost vaccine regimen in the same class as the ALVAC-HIV and AIDSVAX B/E HIV vaccine regimen studied here was shown to be non-efficacious in a subsequent efficacy trial [37], and it is unknown whether the same correlations with non-HIV vaccine-elicited responses would also be seen with other HIV vaccine regimens (differing by, e.g., platform) that are currently in different development pipelines [38,39,40]. Third, given that our analysis focused on immune responses identified as correlates of risk of HIV acquisition in ALVAC-HIV and AIDSVAX B/E vaccine recipients, and that immune correlates may differ by vaccine regimen, different HIV vaccine-induced immune responses may need to be studied for use of baseline immunogenicity predictors with other HIV vaccine regimens. Fourth, global coverage is relatively high for both the tetanus vaccine and the HBV vaccine, estimated in 2023 at 84% for a third dose of vaccine protecting against diphtheria, tetanus, and pertussis and at 83% for three doses of HBV vaccine [41]. Thus, in general, the TT and HBV vaccines might not be the most optimal choices for the non-HIV vaccine baseline immunogenicity predictors of an HIV vaccine. However, given that immune responses wane over time, they may be suitable candidates, depending on factors such as region-specific coverage.

Acknowledging these limitations, we note that our findings are also valuable in that they contribute to the relatively small body of work that has examined whether immune responses induced via one vaccine correlate with those induced via another. With the caveats discussed above, they provide a proof-of-concept that such correlations can be seen for an HIV and a non-HIV vaccine.

## Data Availability

The original data underlying the findings of the study are openly available at the public-facing HVTN page (https://atlas.scharp.org/cpas/project/HVTN%20Public%20Data/begin.view?).

## Supplementary Materials

The following supporting information can be downloaded at https://www.mdpi.com/article/10.3390/v16091365/s1: Table S1. Immune responses in HVTN 097 vaccine recipients selected for assessing correlations with non-HIV vaccine-induced immune responses; Figure S1. Gating strategy for the ICS assay; Figure S2. Correlation plots for (A-D) Month 1 TT-specific CD4+ T-cell responses versus Month 7.5 IgA binding antibody responses against the HIV antigen designated on each y-axis; (E-H) Month 1 TT-specific IgG binding antibody responses versus Month 7.5 IgA binding antibody responses against the HIV antigen designated on each y-axis; (I-L) Month 13.5 HBsAg-specific CD4+ T-cell responses versus Month 7.5 IgA binding antibody responses against the HIV antigen designated on each y-axis; (F-J) Month 13.5 HBsAg-specific IgG binding antibody responses versus Month 7.5 IgA binding antibody responses against the HIV antigen designated on each y-axis; Figure S3. Correlation plots for (A) Month 1 TT-specific CD4+ T-cell responses versus Month 7.5 ADCC responses to 92TH023293FgDneg; (B) Month 1 TT-specific IgG binding antibody responses versus Month 7.5 ADCC responses to 92TH023293FgDneg; (C) Month 13.5 HBsAg-specific CD4+ T-cell responses versus ADCC responses to 92TH023293FgDneg; (D) Month 13.5 HBsAg-specific IgG binding antibody responses versus Month 7.5 ADCC responses to 92TH023293FgDneg; Figure S4. Correlation plots for (A) Month 1 TT-specific CD4+ T-cell responses versus Month 7.5 ICS CD4 responses to 92TH023ENV; (B) Month 1 TT-specific IgG binding antibody responses versus Month 7.5 ICS CD4 responses to 92TH023ENV; (C) Month 13.5 HBsAg-specific CD4+ T-cell responses versus ICS CD4 responses to 92TH023ENV; (D) Month 13.5 HBsAg-specific IgG binding antibody responses versus Month 7.5 ICS CD4 responses to 92TH023ENV; Figure S5. Correlation plots for (A) Month 1 TT-specific CD4+ T-cell responses versus Month 7.5 ICS CD4 polyfunctionality score to 92TH023ENV; (B) Month 1 TT-specific IgG binding antibody responses versus Month 7.5 ICS CD4 polyfunctionality score to 92TH023ENV; (C) Month 13.5 HBsAg-specific CD4+ T-cell responses versus ICS CD4 polyfunctionality score to 92TH023ENV; (D) Month 13.5 HBsAg-specific IgG binding antibody responses versus Month 7.5 ICS CD4 polyfunctionality score to 92TH023ENV. References [16,22,28] are cited in the supplementary materials.
