# Supplementary material for: Non-HIV Vaccine-Induced Immune Responses as Potential Baseline Immunogenicity Predictors of ALVAC-HIV and AIDSVAX B/E-Induced Immune Responses
Source: Viruses. 2024 Aug 27;16(9):1365. doi: 10.3390/v16091365 (PMC11437453; doi:10.3390/v16091365)
Supplement: Supplementary file 1 [file viruses-16-01365-s001.zip › viruses-3113819-supplementary.pdf]

Table S1. Immune responses in HVTN 097 vaccine recipients selected for assessing correlations with non-HIV vaccine-induced immune responses. All adaptive immune responses were previously reported [22]; this table is a subset of the responses shown in S1 Fig in Andersen-Nissen et al. [28].

Timepoint	Assay	Immune Response
Month 7.5	BAMA	1:50 net response (MFI – blank) of IgG bAbs to vector prime insert
Month 7.5	BAMA	1:50 net response (MFI – blank) of IgG bAbs to AE protein boost
Month 7.5	BAMA	1:50 net response (MFI – blank) of IgG bAbs to B protein boost
Month 7.5	BAMA	1:50 net response (MFI – blank) of IgG bAbs to B V1V2 immune correlate
Month 7.5	BAMA	1:50 net response (MFI – blank) of IgG bAbs to AE V1V2 immune correlate
Month 7.5	BAMA	1:50 net response (MFI – blank) of IgA bAbs to vector prime insert
Month 7.5	BAMA	1:50 net response (MFI – blank) of IgA bAbs to Env gp140 immune correlate
Month 7.5	BAMA	1:50 net response (MFI – blank) of IgA bAbs to AE protein boost
Month 7.5	BAMA	1:50 net response (MFI – blank) of IgA bAbs to B protein boost
Month 7.5	ADCC	Area under the curve % granzyme B activity to vector prime insert
Month 7.5	ADCP	Mean phagocytosis score to C Env gp140
Month 7.5	ADCP	Mean phagocytosis score to C V1V2
Month 7.5	ICS	% CD4+ T cells expressing IFN-γ and/or IL-2 in response to vector prime insert stimulation
Month 7.5	ICS	CD4+ T cell polyfunctionality score in response to vector prime insert stimulation

Notes: "Vector prime insert" = 92TH023 gp120; "AE protein boost" = Clade AE A244 gp120; "B protein boost" = Clade B MN gp120; "B V1V2 immune correlate" = Clade B gp70-Case A V1V2; "AE V1V2 immune correlate" = Clade AE A244 V1V2; "Env gp140 immune correlate" = A1.Con gp140; "C V1V2" = 1086 V1V2; "C Env gp140" = 1086 gp140; ADCC = antibody-dependent cellular cytotoxicity; ADCP = antibody-dependent cellular phagocytosis; bAbs = binding antibodies; BAMA = binding antibody multiplex assay; ICS = intracellular cytokine staining.

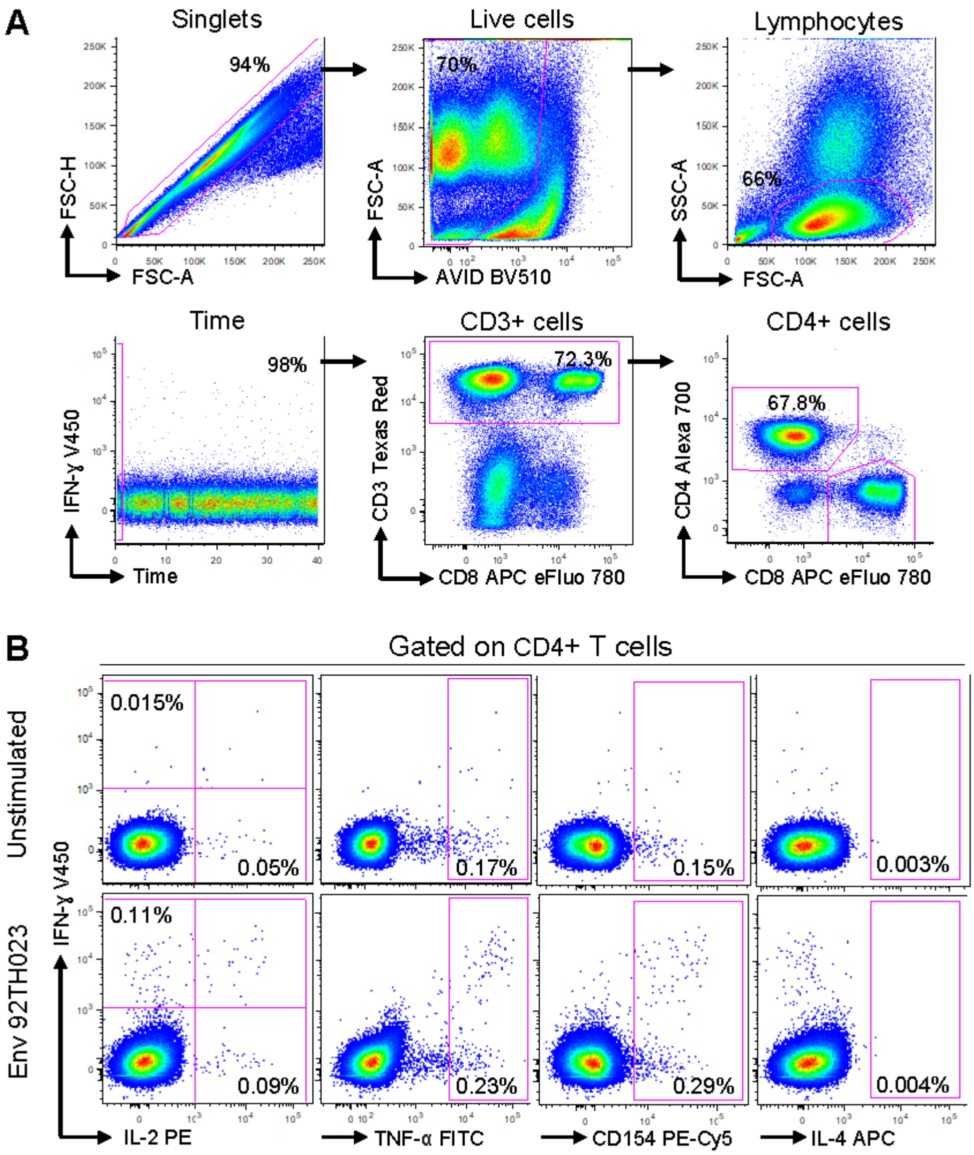

Actually

Figure S1. Gating strategy for the ICS assay. (**A**) Nested gating strategy to identify the CD4+ T cell population. (**B**) Expression of IFN-γ, IL-2, TNF-α, CD154 and IL-4 in CD4+ T cells in unstimulated PBMC (top) and after stimulation with Env 92TH023 (bottom).

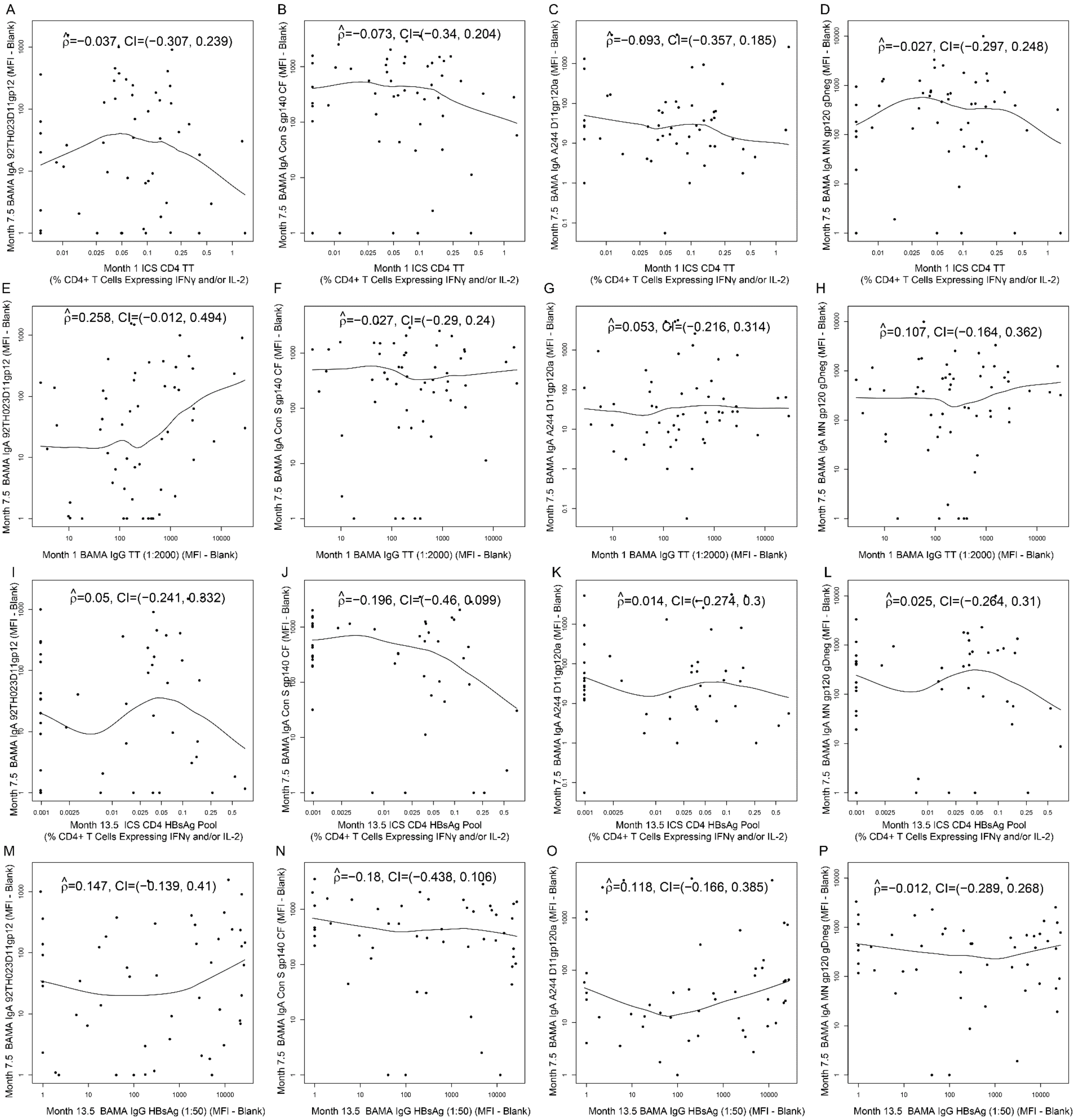

Figure S2. Correlation plots for (**A-D**) Month 1 TT-specific CD4+ T-cell responses versus Month 7.5 IgA binding antibody responses against the HIV antigen designated on each *y*-axis; (**E-H**) Month 1 TT-specific IgG binding antibody responses versus Month 7.5 IgA binding antibody responses against the HIV antigen designated on each *y*-axis; (**I-L**) Month 13.5 HBsAg-specific CD4+ T-cell responses versus Month 7.5 IgA binding antibody responses against the HIV antigen designated on each *y*-axis; (**F-J**) Month 13.5 HBsAg-specific IgG binding antibody responses versus Month 7.5 IgA binding antibody responses against the HIV antigen designated on each *y*-axis. Data are shown for HVTN 097 participants [$T1_{TT\text{-}HIV\text{-}HBV}$ arm only]. ICS CD4 TT: CD4+ T-cell responses (% CD4+ T cells expressing IFNγ and/or IL-2) to TT; BAMA IgG TT: Net IgG binding antibody responses (MFI - blank) to TT; ICS CD4 HBsAg pool: CD4+ T-cell responses (% CD4+ T cells expressing IFNγ and/or IL-2) to HBsAg pool; BAMA IgG HBsAg: Net IgG binding antibody responses (MFI - blank) to HBsAg. Each dot represents one participant. At Month 0, $T1_{TT\text{-}HIV\text{-}HBV}$ participants received TT. At Month 13, $T1_{TT\text{-}HIV\text{-}HBV}$ participants received HBV. At Month 7, $T1_{TT\text{-}HIV\text{-}HBV}$ received the last dose of ALVAC & AIDSVAX B/E. No correlation had FDR≤0.2.

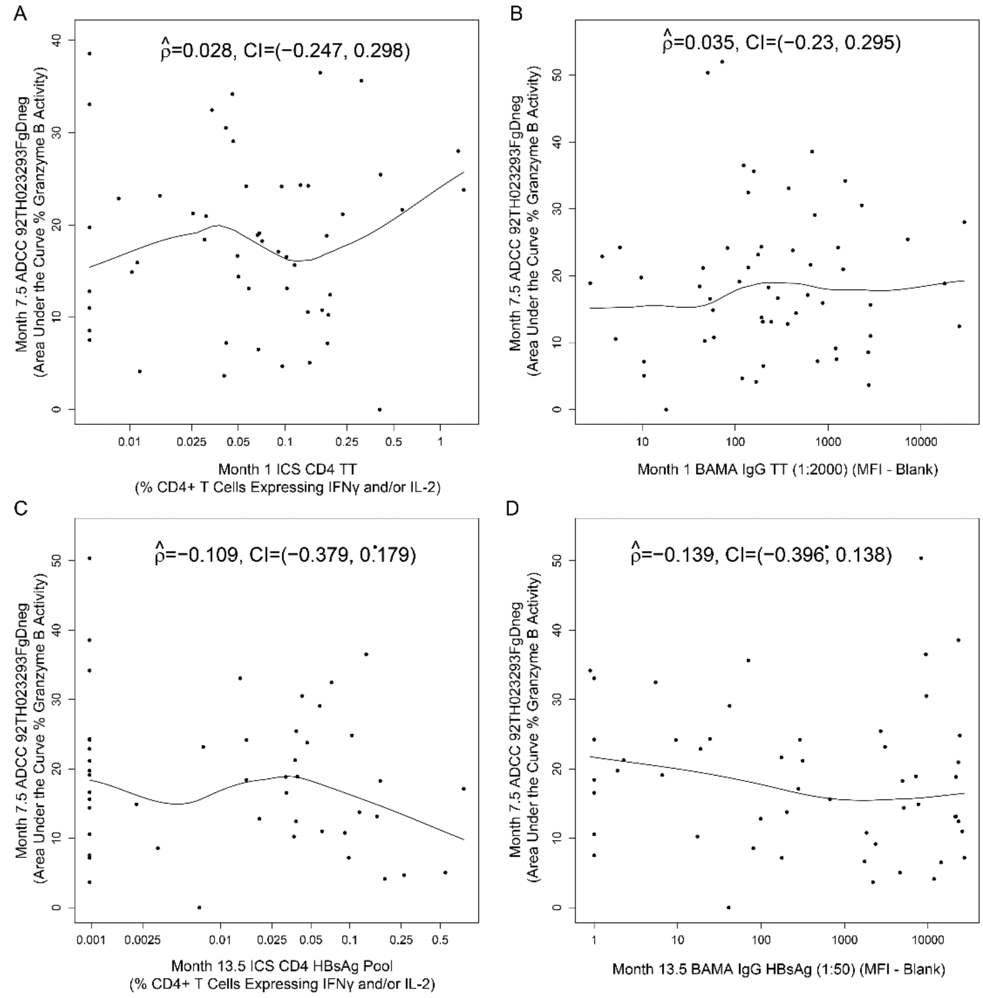

Figure S3. Correlation plots for (**A**) Month 1 TT-specific CD4+ T-cell responses versus Month 7.5 ADCC responses to 92TH023293FgDneg; (**B**) Month 1 TT-specific IgG binding antibody responses versus Month 7.5 ADCC responses to 92TH023293FgDneg; (**C**) Month 13.5 HBsAg-specific CD4+ T-cell responses versus ADCC responses to 92TH023293FgDneg; (**D**) Month 13.5 HBsAg-specific IgG binding antibody responses versus Month 7.5 ADCC responses to 92TH023293FgDneg. Data are shown for HVTN 097 participants [$T1_{TT-HIV-HBV}$ arm only]. ADCC responses to 92TH023293FgDneg: Area under the curve % granzyme B activity; ICS CD4 TT: CD4+ T-cell responses (% CD4+ T cells expressing IFNγ and/or IL-2) to TT; BAMA IgG TT: Net IgG binding antibody responses (MFI - blank) to TT; ICS CD4 HBsAg pool: CD4+ T-cell responses (% CD4+ T cells expressing IFNγ and/or IL-2) to HBsAg pool; BAMA IgG HBsAg: Net IgG binding antibody responses (MFI - blank) to HBsAg. Each dot represents one participant. At Month 0, $T1_{TT-HIV-HBV}$ participants received TT. At Month 13, $T1_{TT-HIV-HBV}$ participants received HBV. At Month 7, $T1_{TT-HIV-HBV}$ received the last dose of ALVAC & AIDSVAX B/E. No correlation had FDR≤0.2.

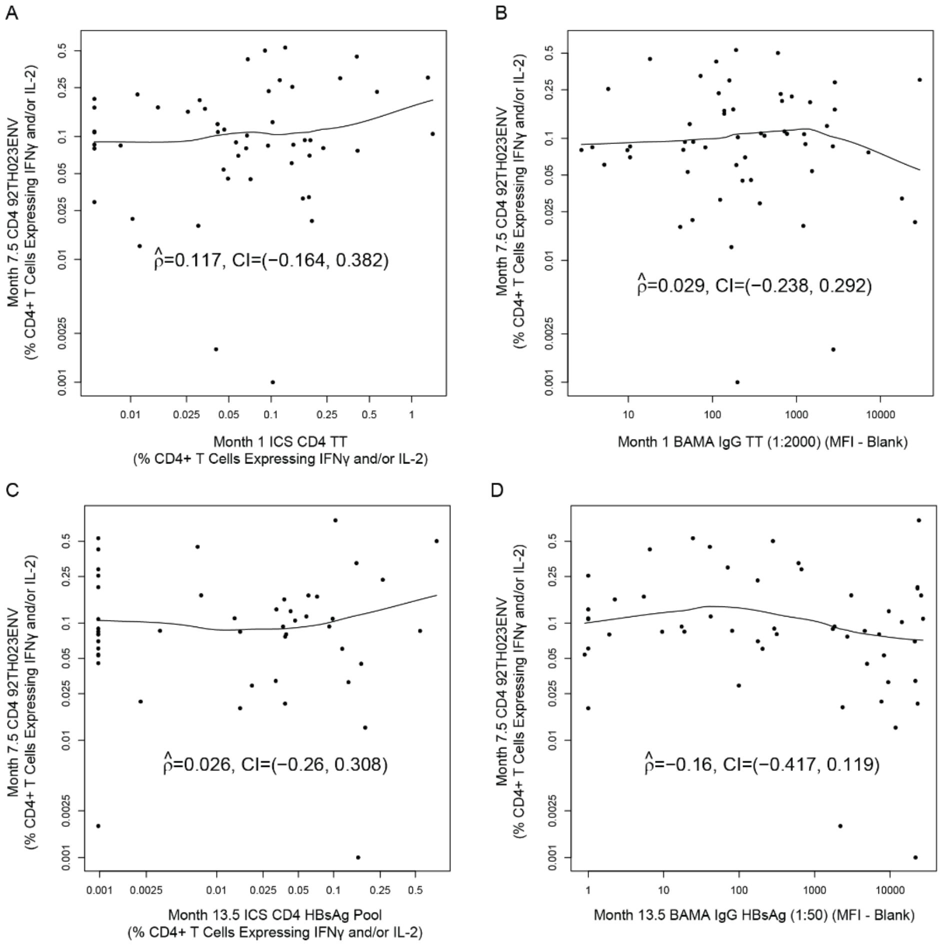

Figure S4. Correlation plots for (**A**) Month 1 TT-specific CD4+ T-cell responses versus Month 7.5 ICS CD4 responses to 92TH023ENV; (**B**) Month 1 TT-specific IgG binding antibody responses versus Month 7.5 ICS CD4 responses to 92TH023ENV; (**C**) Month 13.5 HBsAg-specific CD4+ T-cell responses versus ICS CD4 responses to 92TH023ENV; (**D**) Month 13.5 HBsAg-specific IgG binding antibody responses versus Month 7.5 ICS CD4 responses to 92TH023ENV. Data are shown for HVTN 097 participants [$T1_{TT-HIV-HBV}$ arm only]. CD4 responses to 92TH023ENV: % CD4+ T cells expressing IFNγ and/or IL-2 to 92TH023ENV peptide pool; ICS CD4 TT: CD4+ T-cell responses (% CD4+ T cells expressing IFNγ and/or IL-2) to TT; BAMA IgG TT: Net IgG binding antibody responses (MFI - blank) to TT; ICS CD4 HBsAg pool: CD4+ T-cell responses (% CD4+ T cells expressing IFNγ and/or IL-2) to HBsAg pool; BAMA IgG HBsAg: Net IgG binding antibody responses (MFI - blank) to HBsAg. Each dot represents one participant. At Month 0, $T1_{TT-HIV-HBV}$ participants received TT. At Month 13, $T1_{TT-HIV-HBV}$ participants received HBV. At Month 7, $T1_{TT-HIV-HBV}$ received the last dose of ALVAC & AIDSVAX B/E. No correlation had FDR≤0.2.

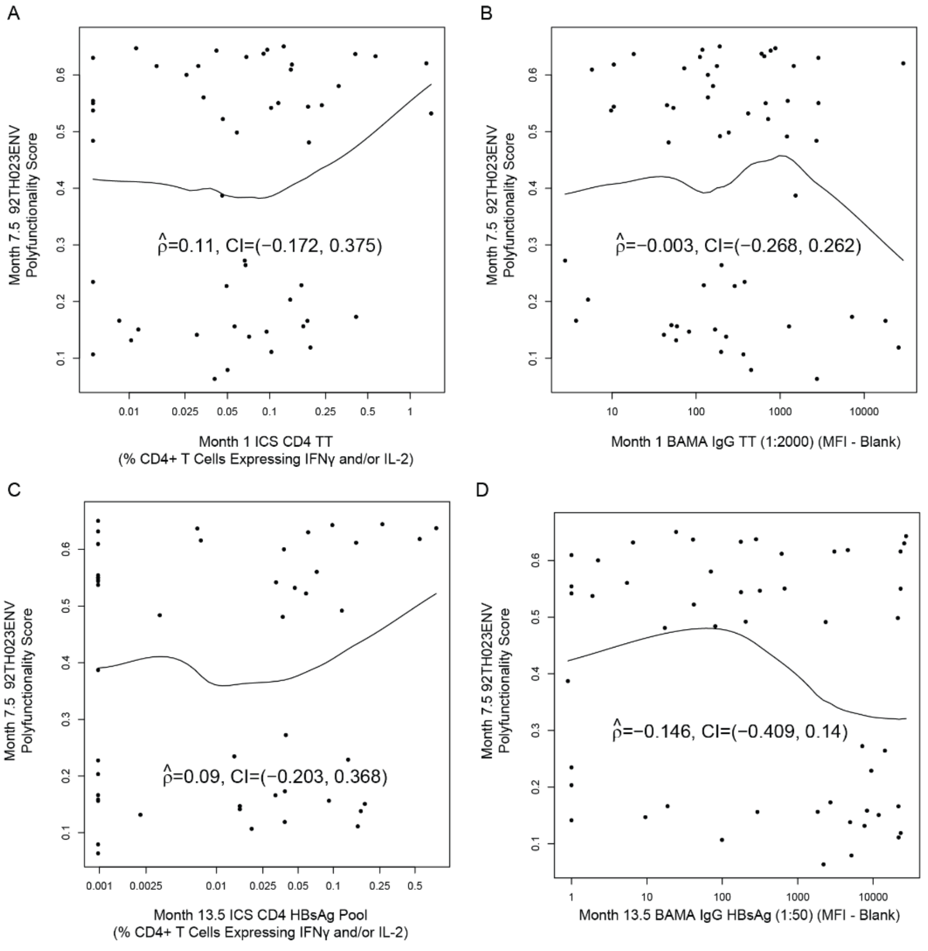

Figure S5. Correlation plots for (**A**) Month 1 TT-specific CD4+ T-cell responses versus Month 7.5 ICS CD4 polyfunctionality score to 92TH023ENV; (**B**) Month 1 TT-specific IgG binding antibody responses versus Month 7.5 ICS CD4 polyfunctionality score to 92TH023ENV; (**C**) Month 13.5 HBsAg-specific CD4+ T-cell responses versus ICS CD4 polyfunctionality score to 92TH023ENV; (**D**) Month 13.5 HBsAg-specific IgG binding antibody responses versus Month 7.5 ICS CD4 polyfunctionality score to 92TH023ENV. Data are shown for HVTN 097 participants [$T1_{TT-HIV-HBV}$ arm only]. CD4 polyfunctionality score (defined in ref. [16]) to 92TH023ENV; ICS CD4 TT: CD4+ T-cell responses (% CD4+ T cells expressing IFNγ and/or IL-2) to TT; BAMA IgG TT: Net IgG binding antibody responses (MFI - blank) to TT; ICS CD4 HBsAg pool: CD4+ T-cell responses (% CD4+ T cells expressing IFNγ and/or IL-2) to HBsAg pool; BAMA IgG HBsAg: Net IgG binding antibody responses (MFI - blank) to HBsAg. Each dot represents one participant. At Month 0, $T1_{TT-HIV-HBV}$ participants received TT. At Month 13, $T1_{TT-HIV-HBV}$ participants received HBV. At Month 7, $T1_{TT-HIV-HBV}$ received the last dose of ALVAC & AIDSVAX B/E. No correlation had FDR≤0.2.
